# Are oligodendrocytes the missing link in Alzheimer’s disease and related dementia research?

**DOI:** 10.1186/s13024-024-00760-6

**Published:** 2024-11-17

**Authors:** Sharyn L. Rossi, Diane E. Bovenkamp

**Affiliations:** BrightFocus Foundation, 22512 Gateway Center Drive, Clarksburg, MD 20871 USA

Oligodendrocytes (OLs) and their lineage progenitor (OPCs) and precursor cells are widely studied and recognized as promising therapeutic targets for multiple neurodegenerative diseases and disorders including multiple sclerosis, spinal cord injuries, traumatic brain injuries, stroke, Parkinson’s disease, ALS, and others. Yet, their role in Alzheimer’s disease and related dementias (ADRDs), despite their complex, multi-factorial nature, remains largely underappreciated and understudied. Mounting evidence supports the role of oligodendrocyte dysfunction in ADRD which begins with their normal homeostatic role during development and non-disease physiological states.

During development, OPCs follow an intricate temporal and spatial pattern as they migrate, proliferate, and differentiate into the myelinating cells of the CNS. While migrating, pools of oligodendrocyte precursor cells and OPCs are distributed throughout the brain and spinal cord. OLs and OPCs release growth factors that play a role in synaptic transmission and participate in synaptic engulfment and remodeling [1]. OPCs are a heterogenous subtype that persist into adulthood and have various (originally considered atypical) functions including neuromodulation through direct synaptic connections with neurons, vascularization and angiogenesis, coordinated interactions with astrocytes in the development and maintenance of the blood brain barrier, and immunomodulatory properties through interactions with microglia. OPCs play a critical role in synaptic remodeling, circuit plasticity, and regenerative mechanisms, proliferating and differentiating in response to an insult or injury. Activated OPCs can undergo oligodendrogenesis to form fully functional mature OLs that participate in the remyelination of disconnected or dysfunctional brain circuits but have also been shown to differentiate into astrocytes and neurons [2]. These responses and functions are tightly controlled by numerous signaling pathways, including Notch and PI3/Akt pathways, that are known to be dysregulated in ADRD [3].

Mature myelin forming OLs are made primarily of lipids and cholesterol and control information processing speed across local neuronal circuits and distal targets using precise modulation to serve appropriate biological functions. As myelination occurs throughout development and into adulthood, the process is heavily influenced by personal experience, lifestyle, and other exposures (the exposome) that modify the transcriptomic signatures of OPCs and OLs. Dynamic shifts in myelin sheath thickness and in the location of nodes of Ranvier (junctions between myelin segments) control information flow and facilitate synchrony between and across neural networks. These networks are required for learning and memory and experience-dependent myelin formation facilitates memory consolidation and recall [4]. In addition to controlling neural activity, OLs provide metabolic support for neurons [5] and interact with microglia and astrocytes to control lipid metabolism, extracellular matrix dynamics, and immune responses [6, 7].

Dysregulation of these OL functions has been implicated in the progression of ADRD and neuroimaging studies have confirmed global changes in myelin integrity for decades. More recently, high profile studies in humans have provided irrefutable evidence that OLs play an integral role in the progression and possibly even the onset of ADRD. SMOC1 is a protein recently discovered in the CSF of people with autosomal dominant AD decades before symptom onset [8]. SMOC1 is almost exclusively expressed in OPCs and OLs in the brain (Single cell type - SMOC1 - The Human Protein Atlas https://www.proteinatlas.org/ENSG00000198732-SMOC1/single+cell+type) and has been implicated in the failure of oligodendrogenesis in Spinocerebellar Ataxia [9]. Furthermore, APOE4, the major risk factor for AD, causes the aberrant deposition of cholesterol in OLs, impairing myelin maintenance and function [10]. Dysfunctional oligodendrogenesis and remyelination in ADRD is likely driven by disease associated transcriptomic changes and the conversion of OPCs and OLs to senescent phenotypes [11]. OLs also contribute and respond to amyloidosis in AD as they accumulate around amyloid plaques, express genes associated with amyloid production [12], and even contribute to amyloid deposition [13, 14]. Perhaps most intriguing is new data released by the Allen Institute showing two AD-associated phases of pathology in postmortem tissue. The first stage consists of loss of myelinating oligodendrocytes and a selectively vulnerable population of somatostatin interneurons which is accompanied by an upregulation of remyelination programs in OPCs that ultimately fail. These findings indicate that OLs are involved at the earliest stages of disease progression, before symptom onset and accumulation of toxic proteins in AD [15].

At a time when the ADRD field is reflecting on “All the Alzheimer’s Research We Didn’t Do” (Piller C. Opinion, https://www.nytimes.com/2024/07/07/opinion/alzheimers-missed-opportunities.html), it is crucial to ensure we anticipate and address any potential pitfalls or gaps as we move forward as a field. Including OLs in ADRD research, particularly in ongoing big data efforts, is essential for a comprehensive understanding of these diseases. Invaluable resources that aim to extensively characterize cell types across various systems and neurodegenerative diseases would lack a fundamental cellular variable whose homeostatic biological functions are inevitably perturbed by the ADRD associated milieu.

It is essential for all researchers focused on ADRD to recognize the significant role OLs and oligodendrogenesis play in neurodegeneration, plasticity, and repair. As key players in maintaining axonal health, modulating synaptic plasticity, and supporting the structural integrity of neuronal circuits, OLs are a suitable and viable therapeutic target for ADRD (Fig. 1). Cross-disease learnings from other neurodegenerative fields can provide valuable insights into the therapeutic potential of OLs and OPCs that can serve as a foundation for advancing ADRD research. Various clinical trials for MS and ALS are aimed at restoring myelin by activating endogenous OPCs, modulating neuroinflammation to create a more conducive remyelination environment, and/or through exogenous transplantation of various stem and progenitor cell populations [16]. Considering these cells as both drivers and modifiers of ADRD pathology will fill knowledge gaps and open new treatment avenues, including targeted senolytics, gene editing, and harnessing the proliferative and differentiation potential of OPCs through noninvasive interventions like focused ultrasound and transcranial magnetic stimulation and possibly even directed differentiation via in situ conversion.

**Fig. 1:**
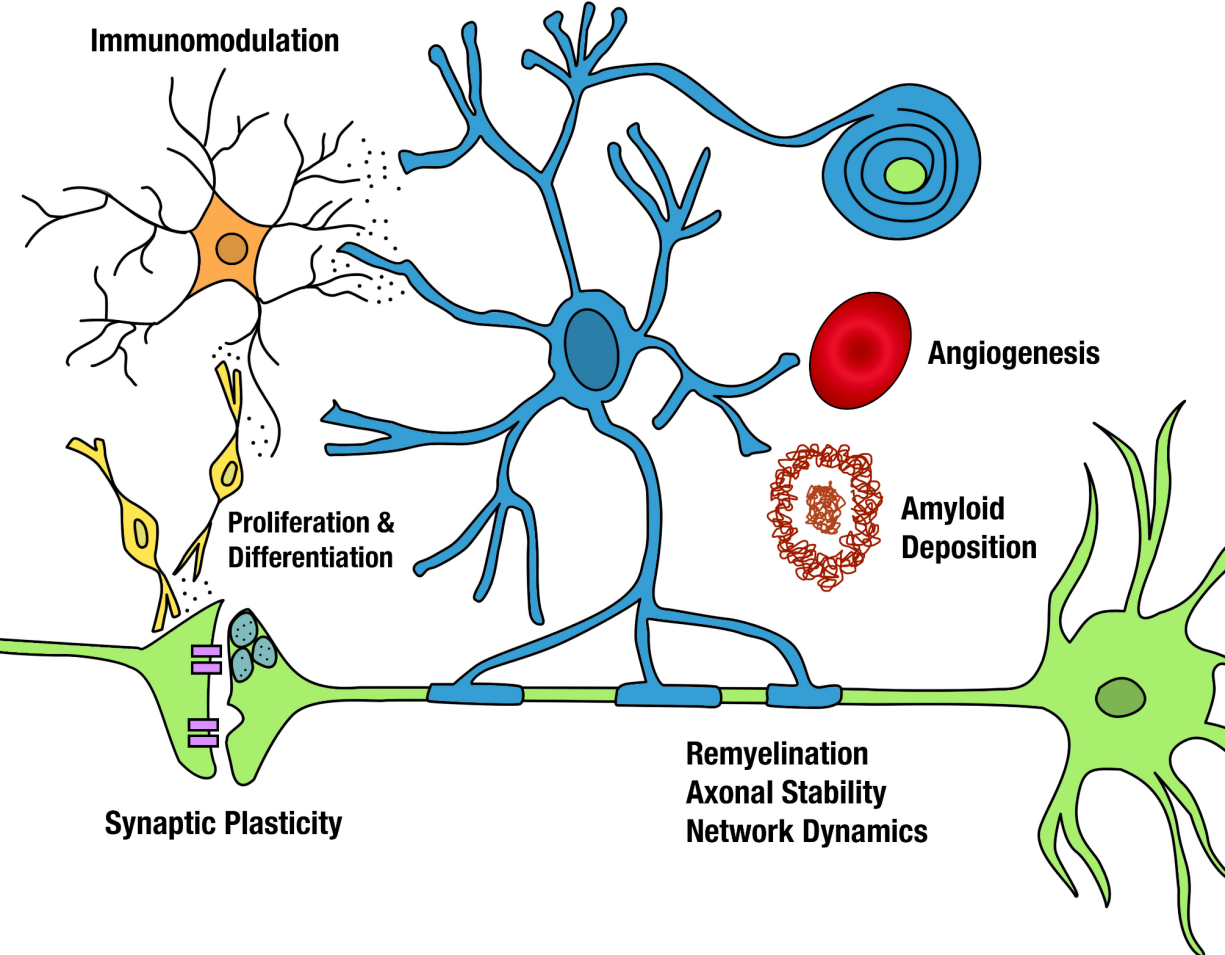
Oligodendrocyte specific therapeutic targets. Mature OLs (blue) and their progenitor OPCs (yellow) are integral to neuronal (green) health and function. Mature OLs maintain axonal stability, providing metabolic support and nutrients to neurons while controlling the rate at which information flows through dynamic changes in myelin structure and shifts in nodes of Ranvier. Both OLs and OPCs interact with other cell types (microglia, orange) and the vasculature (red) as they play a role in angiogenesis and secrete a variety of immunomodulatory cytokines and growth factors. OLs have recently been shown to play a more causative role in amyloid deposition and plaque formation (brown). OPCs are a heterogenous population of cells that persist throughout life and participate in synaptic circuit remodeling. Their proliferative potential could be harnessed to promote oligodendrogenesis resulting in remyelination, neurogenesis to restore functional neurons, or astrogenesis to restore homeostatic astrocytic functions

In the last decade, ADRD research has experienced a significant shift toward viewing and studying dementia through a multisystem holistic lens. Despite multiple reports identifying oligodendrocytes as key mediators of pathological processes in humans and animal models, their full contributions to disease mechanisms and potential as disease modifiers has not been realized. Increased interest in the role OLs play in ADRD can be fueled by increased funding and initiatives focused on oligodendroglial interactions with cell types vulnerable to ADRD pathology. Apparent action items for government, for profit and not for profit funders to include OLs and OPCs in ADRD research could include:

1. Requests that OLs and OPCs be added to all currently active and future initiations of multi-cellular ‘omics,’ systems biology, circuit networks, AI/deep learning, and other techniques/databases to better understand neurodegenerative disease for all organisms, but especially for those studies looking to better understand ADRD risk and progression in humans.
2. Addition of OL and OPC biomarkers to molecular panels researching basic, translational and clinical diagnostic, prognostic and treatment studies in people affected by various ADRD.
3. Addition of OL and OPC biomarkers to systems biology, in situ and in vivo characterization of animal and cellular models of ADRD.
4. ADRD funding agencies should seek out and prioritize projects that include OLs and/or OPCs in their scientific or therapeutic approach. The inclusion of OLs should be strongly considered in large-scale data collection initiatives that compare and contrast cell types involved in ADRD.

## Data Availability

N/A.

## Abbreviations

OL: Oligodendrocyte
OPC: Oligodendrocyte Progenitor Cell
ADRD: Alzheimer’s Disease and Related Dementias
